# THEORIZING HYBRIDITY: INSTITUTIONAL LOGICS, COMPLEX ORGANIZATIONS, AND ACTOR IDENTITIES: THE CASE OF NONPROFITS

**DOI:** 10.1111/padm.12105

**Published:** 2014-07-20

**Authors:** CHRIS SKELCHER, STEVEN RATHGEB SMITH

**Affiliations:** 1 Institute of Local Government Studies (INLOGOV) at the University of BirminghamUK; 2 Executive Director of the American Political Science AssociationWashington, DC, USA

## Abstract

We propose a novel approach to theorizing hybridity in public and nonprofit organizations. The concept of hybridity is widely used to describe organizational responses to changes in governance, but the literature seldom explains how hybrids arise or what forms they take. Transaction cost and organizational design literatures offer some solutions, but lack a theory of agency. We use the institutional logics approach to theorize hybrids as entities that face a plurality of normative frames. Logics provide symbolic and material elements that structure organizational legitimacy and actor identities. Contradictions between institutional logics offer space for them to be elaborated and creatively reconstructed by situated agents. We propose five types of organizational hybridity – segmented, segregated, assimilated, blended, and blocked. Each type is theoretically derived from empirically observed variations in organizational responses to institutional plurality. We develop propositions to show how our approach to hybridity adds value to academic and policy-maker audiences.

## INTRODUCTION

The purpose of this article is to demonstrate how the Institutional Logics Approach (ILA) can introduce theoretical and conceptual clarity into the discussion of ‘hybridity’ in the public administration and nonprofit fields, and open up a promising research agenda on a topic of considerable academic and practical significance. The development of a theory of hybridity applicable to the public and nonprofit sectors is well overdue. Global public management reforms and changes in governance have stimulated the emergence of a variety of organizational forms to which the label ‘hybrid’ is often attached. These include public–private partnerships, contracted-out service delivery structures, quasi-autonomous agencies, and user-managed public facilities, collaborative forums of various types, social enterprises, and systems of network governance (e.g. Kickert 2001; Koppell 2003; Skelcher 2005; Sørensen and Torfing 2009; Smith 2010).

Depending on the extent to which national administrative cultures have moderated global New Public Management (NPM) trends, these new forms have either replaced the archetypical, politically headed public bureaucracies as the primary arena for policy development and programme delivery, or sit alongside them connected through diverse formal and informal mechanisms. Additionally, some public organizations that pre-date NPM can be conceived as hybrids. Public enterprises, for example, are profit-seeking but operate within the public realm. Even government departments might be deemed to be hybrids where they exhibit the characteristics of Weberian bureaucracy and new managerialism (Meyer *et al*. 2014).

In the nonprofit sector, hybridity typically refers to the complex organizational forms that arise as voluntary, charitable, and community organizations confront differentiated task, legitimacy, or resource environments. An important early contribution is Minkoff’s (2002) study of nonprofits that combine the distinct organizational forms associated respectively with service provision and political advocacy. There is also a significant literature on the effects of contracting by government for delivery of public services, and specifically the tensions within a nonprofit between servicing government’s requirements and sustaining the original social mission, a debate often framed in terms of hybridization (e.g. Evers 2005; Mullins 2006; Binder 2007). Hybridity has also emerged as a consequence of nonprofits’ search for new sources of revenue to fund their core mission, for example by creating for-profit subsidiaries to generate revenue for the parent body (e.g. Cooney 2006; Smith 2010).

In the light of this discussion, one might be inclined to agree with Brandsen *et al*. (2005, p. 758) that ‘hybridity [is] an inevitable and permanent characteristic’ of the nonprofit sector. They argue that the domains of market, state, and civil society are no longer able to be classified in a mutually exclusive way, thus making it difficult to create an unambiguous definition of the nonprofit sector. ‘In light of these difficulties’, they continue, ‘… [i]t is questionable whether further, more refined typologies based on structural characteristics of domains and organizations could do justice to the developments that we are currently seeing’ (pp. 758–59). A similar argument could be made in relation to the public sector, where the clarity of the boundary between public and private purposes has been eroded by marketization. If structural concepts no longer offer a guide, Brandsen *et al*. argue, then the focus of scholarship should be to explore whether there are particular rationalities that shape actors’ responses to hybridity.

Although we do not agree with the determinism implied by Brandsen *et al*.’s ‘inevitability’ hypothesis, we are sympathetic to the notion that rationalities are at play. Yet this leaves the problem of conceptualizing these rationalities and theorizing how they are generative of hybridity. This is the challenge we address. Our argument is that hybridization arises from a plurality of rationalities – which we term ‘institutional logics’ (e.g. Mullins 2006; Sacranie 2012; Meyer *et al*. 2014; Pache and Santos 2013). We view hybridization as a process in which plural logics and thus actor identities are in play within an organization, leading to a number of possible organizational outcomes. It is the purpose of a theory of hybrids to explain these matters and thus to generate testable propositions.

To date, the public administration and nonprofit literature has under-theorized the concept of hybridity, employing it largely as a descriptor for an organization comprising multiple features of the market/hierarchy/network or state/business/community triptychs. Some initial steps have been taken towards developing a theory of hybrids (e.g. Brandsen *et al*. 2005; Hasenfeld and Gidron 2005; Billis 2010; Christensen and Lægreid 2011; Jäger and Schröer 2013), but the field still lacks a clear theoretical foundation that can explain what it is that creates a hybrid, whether different forms of hybrid emerge in different situations, and if so what consequences arise. In the organizational studies field, however, there has been considerable discussion of these issues.

The first objective of our article, therefore, is to establish the extent to which any of these organizational theories might provide a basis for explaining hybridity in public and nonprofit organizations. Our review leads us to propose that the institutional logics approach can help to fill the gap since it firmly locates the study of hybridity within a well-developed theoretical tradition (e.g. Alford and Friedland 1985; Kraatz and Block 2008; Thornton *et al*. 2012). This material is covered in the next two sections. The following section then addresses our second objective, which is to formulate a theory of hybridity. We propose that a public or nonprofit hybrid is an organization that incorporates plural institutional logics and where, as a result, organizational members confront multiple identities. By hypothesizing different responses to such institutional plurality, we are able to isolate different types of hybridity.

This theory increases our analytical purchase on the internal dynamics of nonprofit and public organizations in relation to wider structural changes in the governance of societies, as some public administration and nonprofit studies are now illustrating (e.g. Meyer and Hammerschmid 2006; Reay and Hinings 2009; Mullins *et al*. 2012). Our final objective, dealt with in the conclusion to the article, is to identify ways in which this theoretical formulation could frame a research agenda. We offer initial hypotheses regarding the interaction between the variables in the theory, supported by the empirical literature now emerging on these issues.

## APPROACHES TO CONCEPTUALIZING AND THEORIZING HYBRIDITY

*Hybridity* and *the hybrid organization* are slippery concepts with inexact empirical referents; Ménard observes that they are a ‘collection of weirdos’ (Ménard 2004, p. 3). Williamson’s (1996) transaction cost economics (TCE) framework is an essential starting point. He proposes three TCE governance structures: market; hierarchy; and the hybrid, an intermediate form. In theoretical terms, hybrids trade off some of the price incentives and actor autonomy found in market governance for the administrative control and coordination provided by hierarchy. But here, immediately, a problem exists. While market and hierarchy are mutually exclusive, the hybrid does not form a discrete third category. Instead, it manifests itself along a continuum of which market and hierarchy are the discrete end points. Consequently we can separate the concepts at a theoretical level, but we are left with the empirical problem of delimiting where hybridity ends and market or hierarchy starts. The multiple possible manifestations of hybridity sit uneasily against the unitary definitions of market and hierarchy. If hybridity is everything except market and hierarchy, and these two concepts themselves are the limiting cases of a continuum, then the conclusion is that the majority of empirical case will *de facto* be hybrids. This rather reduces the contribution of the concept and its empirical usefulness in distinguishing governance forms other than in purely theoretical TCE analysis.

The public administration and nonprofit literature overcomes this conceptual problem in two ways. One approach is to treat hybrids as if they were a discrete third category. This is exemplified in Koppell’s analysis of Fannie Mae and Fannie Mac, Federal agencies operating in the housing market that are ‘created by … government … to address a specific public policy purpose [and]… owned in whole or in part by private individuals or corporations and/or generat[ing] revenue to cover [their] operating costs’ (Koppell 2003, p. 12). He argues that these are hybrids because they deliver public policy, but have a corporate status that gives them greater autonomy than would be possible if they were constituted as a government department.

The other approach builds on the work of Powell (1990), Borys and Jemison (1989), and others by translating the TCE formulation into a state/market/community or government/business/nonprofit triptych (the exact terminology varies from author to author). In this literature hybridity is not defined as a category *in* the triptych (as in Koppell’s approach), but as the descriptor *of* a combination of two or more of these categories. For example, social enterprises that embody both nonprofit and for-profit elements have been defined as hybrids (e.g. Dees 1998; Evers 2005; Alter 2007). But while these two approaches solve the definitional problem, they do so at the cost of the solid theoretical base that TCE provides.

The public administration and nonprofit literatures outlined above locate hybridity with reference to forms of societal governance, but what if we were to consider the problem of hybridity as manifest within an individual organization? Here we can draw on the organizational design literature, and particularly Mintzberg (1993), who proposes five ideal-typical designs: simple structure where coordination is achieved through direct supervision by the strategic apex; machine bureaucracy in which there is a high degree of specialization, formalization, and standardization; professional bureaucracy in which the complexity of the tasks undertaken by the organization is such that it requires delegation to skilled front-line workers; divisionalized form characterized by multi-functional and semi-autonomous operating units; and the adhocracy in which work teams form and reform in order to solve problems that arise from a complex and dynamic task environment.

Mintzberg argues that empirically identifiable organizational forms typically exhibit various combinations of these ideal types, and thus constitute hybrids. As in TCE, these combinations arise from the theoretical advantages offered by combinations of the ideal types. For example, a strong ‘pull’ by simple structure and adhocracy is hypothesized to produce an entrepreneurial adhocracy in which small self-organizing teams are coordinated by an overall manager. This approach overcomes the problem TCE faces in defining hybridity at the empirical level, but lacks a theory of agency to explain how the relative ‘pull’ (as Mintzberg describes it) exerted by each of the five basic types arises and is generative of a hybrid form.

Archetype theory begins to address these problems. It posits that institutionally legitimated interpretive schemes operate within organizational fields, and shape the orientation of actors towards particular conceptions of organizational design, practice, and task. An archetype, then, is ‘a set of structures and systems that consistently embodies a single interpretive scheme’ (Greenwood and Hinings 1993, p. 1055). Greenwood and Hinings argue that these interpretive schemes arise from professional associations, government, and other sector-wide bodies, and are reinforced by normative frameworks (for example, regulatory requirements). Organizational redesign occurs as a result of changes in the environment affecting that organizational field, mediated by key field-level actors and intra-organizational processes. For example, professional associations act as institutional entrepreneurs who promote new practices. However, new designs will tend to remain in a ‘design track’ or path such that they evolve within the broad parameters of the prevailing archetype. Exceptionally, there may be ‘design excursions’ outside the track, including hybrid forms that emerge between archetypes, but these are regarded as unlikely to survive the normative and functional imperatives operating in the field.

## USING THE INSTITUTIONAL LOGICS APPROACH TO THEORIZE HYBRIDS

Archetype theory’s somewhat conservative and functionalist orientation (Kirkpatrick and Ackroyd 2003) theorizes hybrids as short-lived exceptions to the norm, a conclusion that runs against the prevailing view in the public administration and nonprofit literature. What it does do, however, is return us to Brandsen *et al*.’s observations about the place of rationalities in explaining hybrid forms. It does this by connecting organizational designs to broader normative frames by way of the agency of actors.

We develop this insight by utilizing the institutional logics approach (ILA). ILA adopts a similar perspective on the connectivity between organizational form, normative frames, and individual agency, but sets this within a more substantial social theory. It also enables us to theorize hybridity as a non-exceptional but not necessarily universal event, thus overcoming the weaknesses in both archetype theory (which underplays the incidence of hybridity) and TCE (where hybridity can be considered to be the norm since it is everything except the limiting cases). Finally, ILA contains a theory of agency – it explains how hybridity arises – thus providing a component that is often missing in the public administration and nonprofit literature.

ILA developed within the wider field of institutional theory as a way of explaining the interactions between normative societal structures, organizational forms, and individual behaviour. The central argument is that:

Each of the most important institutional orders of … society has a central logic – a set of material practices and symbolic constructions – which constitutes its organizing principles and which is available to organizations and individuals to elaborate. … These institutional logics are symbolically grounded, organizationally structured, politically defended, and technically and materially constrained, and hence have specific historical limits. (Friedland and Alford 1991, pp. 248–49)

These logics give identity and meaning to actors. However, the contradictions inherent in a plurality of logics provide the space within which actors can elaborate or manipulate these cultural and material resources, thus transforming identities, organizations, or society (Thornton and Ocasio 2008; Greenwood *et al*. 2010). Institutional logics, therefore, are supra-organizational and abstract, but become observable in the concrete social relations of actors who utilize, manipulate, and reinterpret them.

At its heart, the institutional logics approach comprises a theory with five core elements. First, society is understood as an inter-institutional system comprising theoretically distinct normative structures, each with their own logic. These institutional orders are derived by Alford and Friedland (1985) from their social theory, and in their Western-centric formulation comprise: market capitalism, state bureaucracy, democracy, nuclear family, and Christian religion. Subsequently Thornton *et al*. (2012), currently the main proponents of ILA, have offered a less contextually specific and more inclusive set of institutional sectors: market, state, community, family, religion, profession, and corporation. Each sector has its own logic or rationality: ‘socially constructed, historical patterns of cultural symbols and material practices, assumptions, values and beliefs by which individuals produce and reproduce their material subsistence, organize time and space, and provide meaning to their daily activity’ (Thornton *et al*. 2012, p. 51). These are expressed through their distinctive and ideal-typical sources of legitimacy, authority, and identity (Table 1).

**Table 1:** The ideal-typical logics of institutional orders

Variables	Institutional orders						
	Family	Community	Religion	State	Market	Profession	Corporation
Source of legitimacy	Unconditional loyalty	Unity of will; belief in trust and reciprocity	Importance of faith and sacredness	Democratic participation	Share price	Personal expertise	Market position
Source of authority	Patriarchal domination	Commitment to community values and ideology	Priesthood charisma	Bureaucratic domination	Shareholder activism	Professional association	Board of directors/top management
Source of identity	Family reputation	Emotional connection; ego-satisfaction and reputation	Association with deities	Social and economic class	Faceless	Association with quality of craft	Bureaucratic roles

*Source:* Adapted from Thornton *et al*. (2012, p. 73).

A consequence of theorizing institutions as plural is to make the analysis non-deterministic, and instead focus attention on the relationships between the institutional orders, the organizations located within those orders, and the individuals within those organizations. Friedland and Alford’s article explores the relationship between these three interdependent but relatively autonomous levels, in which they see ‘individuals competing and negotiating, organizations in conflict and coordination, and institutions in contradiction and interdependency’ (Friedland and Alford 1991, pp. 240–41).

This leads to the second feature of the theory, which is that agency is enabled through the plurality of logics. The literature in organizational sociology (e.g. Kraatz and Block 2008; Dunn and Jones 2010; Sanders and McClellan 2014) and nonprofit studies (e.g. Mullins 2006; Binder 2007; Pache and Santos 2013) increasingly recognizes the multiplicity of logics bearing on organizations and individuals, even if one may be dominant, and the different ways in which individuals and groups respond. In line with ILA’s non-deterministic approach, the agency of actors at the micro-level is theorized to affect the way in which logics are managed and resolved within organizations and in turn their construction at the societal level. Thus, we have a world of situated actors whose agency is enabled and constrained by the prevailing institutional logic(s), and who creatively respond by adapting organizational forms in order to better fit a complex institutional environment (Pratt and Foreman 2000; Kraatz and Block 2008).

Actors exercise agency as they make sense of the relationship between the normative expectations of an institutional logic and the organizational context in which they find themselves. Thus the third feature of the theory is that organizations as social entities are a medium through which the logics of sectors interact with the agency of actors. Organizations are a way of mobilizing collective effort in a particular context, and thus provide a focus for the expression of an institutional logic in terms of the identity, discourse, and normative framing of its members or stakeholders (Meyer and Hammerschmid 2006; Saz-Carranza and Longo 2012). Indeed, one of the methods scholars employ to identify institutional logics is to analyse inductively the texts and expressions of individual identity generated by actors. This is significant for our discussion of hybrids because, as we explore below, they can be conceptualized as a contingent settlement between plural institutional logics within one organizational entity.

Fourth, institutional logics have both material and cultural or symbolic components (Thornton and Ocasio 2008; Reay and Hinings 2009). Since an institutional logic provides a normative framing, it allocates worth or value differentially and thus affects the material circumstances of individuals and groups. For example, the market logic will emphasize accumulation of personal wealth and income differentials while the religious logic may favour modesty of means and personal charity. But at the same time, the process of identification is a cultural mechanism through which actors gain an allegiance to the particular symbols of a logic. Finally, the theory emphasizes historical contingency (Friedland and Alford 1991). The dominant type of logic, its manifestation in an organizational form, the space it provides for agency, and its material and symbolic aspects are all subject to a particular spatial and temporal setting. This can be seen in the changing relationship between market and state logics in those nations where NPM has been widely adopted (e.g. Evers and Laville 2004). So Friedland and Alford’s theoretical assertion of historical contingency is important in the context of the analysis of hybrids because it introduces an explicit concern with the dynamic relational aspects of institutional logics.

## PLURAL INSTITUTIONAL LOGICS AND HYBRID ORGANIZATIONAL FORMS

It is the plurality of institutional logics and their availability for utilization by actors within organizations that makes this theory highly relevant to the study of hybridity. Rather than conceptualizing hybrids descriptively as entities that somehow combine different sectoral characteristics or organizational forms, a theoretically richer approach is to propose that they are carriers of multiple institutional logics. This insight is reflected in recent work by nonprofit scholars, including Knutsen (2012, p. 988), who observes ‘… a complex picture of institutional logics embodied in the nonprofit sector’.

A number of nonprofit scholars have begun to theorize the organizational consequences of plural institutional logics. For example, Haveman and Rao (2006) study mutual savings organizations to explain the mechanisms that blend different institutional logics to create various hybrid types, while Galaskiewicz and Barringer (2012) assess whether social enterprises that combine a charity and business logic should present these in an ambiguous blended form or as segmented, pure types facing different stakeholders. There is much less in the public administration literature, although this is now becoming of some interest (e.g. Saz-Carranza and Longo 2012). However, the literature lacks a generic model through which the presence of plural institutional logics is connected to different forms of hybridity.

Our approach addresses this problem. We propose five types of hybrids based *a priori* on particular combinations of institutional logics: *segmented*, *segregated*, *assimilated*, and *blended* hybrids are structural ways of accommodating institutional pluralism within the organization; the *blocked* hybrid represents a situation where the organization is unable to resolve the contradictions between different logics (Table 2). We do not claim that these are the only possibilities; they offer some empirically feasible organizational responses to plural institutional logics. Their purpose is to illustrate how the theory might be developed, and to provide a basis for indicative hypotheses (set out in the conclusion to this article) that explain how particular types of hybrid may emerge. The five types are informed and illustrated by the literature on nonprofit hybrids because there is a greater wealth of material in this field. However, we do not consider the five types of hybrids to be specific to one sector or country, although this is an issue that future research might address.

**Table 2:** Theoretical nonprofit hybrid types

Hybrid type	Characteristics	Example	Relevant institutional logics
Segmented	Functions oriented to different logics are compartmentalized within the organization	A nonprofit service agency funded by donations runs a small for-profit activity recycling clothing to fund one-off innovative projects; this forms one unit within the organizational structure	Compartmentalizing the market logic of small-scale revenue generation in the wider context of the professional logic of expert decision-makers
Segregated	Functions oriented to different logics are compartmentalized into separate but associated organizations	A membership nonprofit values inclusiveness and openness in decision-making, its board meetings being open to all members; its affiliated foundation has an exclusive board of philanthropists meeting in private whose mission is to generate large donations for the nonprofit	Compartmentalizing the corporate logic of fundraising from high worth individuals from the democracy logic of the nonprofit’s members
Assimilated	The core logic adopts some of the practices and symbols of a new logic	A nonprofit has adapted its communications to speak the language of performance targets in order to gain legitimacy with external funders, but retains a strong paternalistic approach to staff management	Elements of market logic assimilated into family logic, but family logic retains dominance
Blended	Synergistic incorporation of elements of existing logics into new and contextually specific logic	A nonprofit with government, philanthropic, and earned income provides training for the disadvantaged through a restaurant	A new social enterprise logic emerges from elements of state, community, and corporate logics
Blocked	Organizational dysfunction arising from inability to resolve tensions between competing logics	A nonprofit founded by a small group of individuals retains a strong norm of informal, collective decision-making, yet is required through its contracts with government to adopt a conventional corporate structure with formal hierarchical roles	Irresolvable contradiction between democracy logic of the founders and state logic of the funders

### Compartmentalizing logics through segmented and segregated hybrids

Compartmentalization can be observed where the organizational structure is such that its constituent logics relate independently to its institutional constituencies (Pratt and Foreman 2000; Kraatz and Block 2008). A common rationale for compartmentalization in the nonprofit world is where an organization has to manage the relationship between two logics, for example between a community logic emphasizing service delivery and a market logic reflecting the need to generate income. Tensions between logics are apparent in the public sector, for example in public–private partnerships (Skelcher and Sullivan 2008). Segmented and segregated hybrids are variations on a theme of compartmentalization. The *segmented* hybrid is characterized by compartmentalization within a single organization. Here, the constituent functions or units reflect different institutional logics. In contrast, the logics in the *segregated* hybrid have a greater degree of insulation from each other by virtue of being located within distinct but interconnected organizations.

Cooney (2006) offers an example of a highly developed *segmented* hybrid, a major US social services corporation with tax-exempt status that generates 80 per cent of its revenue from its own internal businesses, retailing donated clothing and packaging products for corporate clients. Within this single organization there is ‘a highly separated, multidivisional form, where the social service division and the two business divisions are each independently run, with separate budgets, goals and operating procedures’ (2006, p. 147). In contrast, the *segregated* hybrid can take a number of forms (Smith 2010). These include the affiliated foundation, a charity designed to raise substantial private donations for the parent nonprofit; and the for-profit subsidiary, which is designed to generate profits that can then be passed to the parent organization. Community Wealth Ventures, a for-profit subsidiary, of the nonprofit organization, Share our Strength, is one of the more prominent examples, others being charity shops, cafes, and even an architectural design firm. Franchising from a national body to local organizations offers another mechanism for segregation (Oster 1996).

Segmentation is likely to transform into segregation as the scale and commercialization of fundraising increases, a process that Billis (2010, p. 61) terms ‘organic hybridization’. This shift is also stimulated by charity and tax legislation and contracting with government agencies (Smith 2006). Both promote a professional logic (Hwang and Powell 2009) that may more easily be managed through the location of such contracted-out provision in a separate unit or subsidiary organization. For example, some nonprofit activities, such as food banks and other emergency food programmes, are supported entirely through donations, and thus the organization is likely to be able to manage this tension between logics through segmentation within the existing organization. The service programmes of other nonprofits are highly dependent upon commercially generated income or public contracts, and thus segregation is a more likely solution.

### Assimilated hybrid

In the *assimilated* hybrid, the core or original logic remains but the organization adopts some of the practices and symbols of a new logic. Rather than compartmentalizing the logics, a selective incorporation of elements of each occurs. Pache and Santos (2013) show how work integration social enterprises selectively connect elements from competing social welfare and market logics in order to gain legitimacy with their stakeholders. In a similar vein, Reay and Hinings (2009) and Townley (2002) illustrate how assimilation can arise as a strategy of resistance to the incursion of a new institutional logic that is authoritatively promoted by an external stakeholder. In these cases, the organization reflects the expectations of the new logic in terms of its structure, symbols, and language, but in its day-to-day practice continues to operate in line with its institutional origins.

This may involve a certain level of duplicity, where the organization presents in terms of the expectations of one logic but otherwise operates in terms of another. For example, in social welfare delivery nonprofits, the principal (e.g. a government funder or the nonprofit CEO) has specific expectations about the delivery of services and the agent, such as a social worker with professional autonomy, has responsibility for implementing the programmes. In this situation, the strategic apex of the organization will conceivably be representing itself to government in terms of a state institutional logic of performance targets, financial accountability, and procedural regularity, while in practice social workers may be following a professional logic that emphasizes the personalization of the service (Lipsky 2010). Another example common in many nonprofit organizations with a faith affiliation is to display religious symbols on the walls. However, the actual services provided by these organizations may be entirely secular (Smith and Sosin 2001).

### Blended hybrid

The *blended* hybrid is one in which the logics evolve into a novel and contextually specific form, in a way that enables them to ‘forge durable identities of their own’ (Kraatz and Block 2008, p. 251) or, as Jäger and Schröer (2013, p. 5) term it, ‘functional solidarity’. This is similar to Pratt and Foreman’s (2000) concept of integration, in which the multiple identities generated by institutional pluralism are resolved through the incorporation of their synergistic elements into a new singular identity. Empirical examples are provided by Minkoff (2002), who reports on the blending of identity-based service provision and political action logics in a nonprofit’s core identity, thus bridging across the legitimacy of existing organizational forms and enhancing the potential for innovation. Similarly, Knutsen (2012) talks about nonprofits developing adapted logics from the historically contingent interactions between market, government, and democracy logics (see also Binder 2007).

Other examples of blending are found in social enterprises that combine or merge different sectoral elements. For instance, Billings Forge Community Works in Hartford, Connecticut operates a restaurant staffed with disadvantaged youth and young adults. The organization receives government contracts, income from the restaurant, and substantial foundation funding. So its model is in some respects a unique mix of three logics: the restaurant has a market logic, but it reflects the community mission of the Billings Forge and the priorities of government since the restaurant also receives public funding for its training programmes. Similarly, the Erie Canalway National Heritage Corridor Commission and the Erie Canalway Heritage Fund is a public–private partnership which brings together public funds and engagement and philanthropic donations to support the Erie Canal, a blended model found in other parks and recreation organizations.

### The blocked hybrid: irreconcilable tensions between logics

The *blocked* hybrid refers to a situation where the inherent tensions between logics cannot be resolved or managed, leading to organizational dysfunction. Nonprofits frequently originate as informal collectives with a high degree of inclusive decision-making and relatively flat organizational structures, informed by the collectively oriented institutional logics of community or religion (Smith and Lipsky 1993; Oster 1996). The imperatives of growth, government funding, and external legitimacy emphasize institutional logics of the state, corporations, and profession, and thus expectations that such organizations will adopt more corporate and hierarchical forms of decision-making. The tensions between these original and externally imposed institutional logics sometimes provoke serious disquiet amongst organizational members, leading to stalemate.

For example, a community health agency in the USA was started by a group of dedicated community volunteers. They remained as board members after the agency was formally incorporated as a nonprofit. However, the newly appointed executive director brought a growth imperative and a more entrepreneurial style to the organization. This generated a tension between the board – whose identity was located in the originating community logic – and the executive director – whose identity was informed by corporate and market logics. As a result, the agency had great difficulty moving ahead with its strategic priorities.

A further example pertains to the consequences of organizational growth. As nonprofits grow, they tend to become more diffuse programmatically, especially if funding has been substantial. When growth reaches a plateau or starts to decline, nonprofit executives are likely to scrutinize different programmes for their financial viability and sustainability. This emphasizes a market logic, and may lead to cutbacks in smaller programmes or those that are poorly linked to the mission-based priorities of the organization. These programme cutbacks can be controversial, prompting staff dissension and possibly exit from the organization. Chambre (2002), for example, found that the closure of nonprofit AIDS service organizations was related in part to the ossification of organizational practices whereupon agencies failed to adapt to changing circumstances.

Similarly, Seibel (1996) argued that nonprofit organizations in two service categories – domestic violence programmes and employment programmes for the disabled – could be characterized as ‘low-performance, high-persistence’ because funding agencies were unable or unwilling to push the organization to adapt their programmes to be effective. A faith-related example is the effort by some municipalities, such as the City of San Francisco, to require that all of their nonprofit contractors have human resource benefits for same-sex couples, a position opposed by some faith-related service contractors. This conflict has then led in some circumstances to lawsuits and fractious debate (Lattin 1998). In a broader sense, this faith-related example typifies the potential for conflict between the government norms of equity and the primacy placed by nonprofits on being responsive to their community of interest (Smith and Lipsky, 1993).

## PLURAL INSTITUTIONAL LOGICS AND MULTIPLE ACTOR IDENTITIES

To see plural institutional logics solely as giving rise to such hybrid organizational forms would be seriously to underplay the potential of ILA. It is important to recognize that the underpinning social theory connects societal, organizational, and individual levels. It theorizes actors as situated, taking identity and meaning from the normative frames supplied by institutional logics, but also reinterpreting and reshaping them through their contingent agency within an organizational context (Zilber 2002). Consequently the existence of plural institutional logics, especially when the relationship between them is changing, is potentially generative of political contestation, a point that Friedland and Alford (1991, p. 256) emphasize:

Some of the most important struggles between groups, organizations, and classes are over the appropriate relationships between institutions, and by which institutional logic different activities should be regulated and to which categories of person they apply.

This contestation results from the way in which institutional logics structure the rules of the game, and thus the distribution of political resources. As Kraatz and Block (2008, p. 243) observe, ‘an organization confronting institutional pluralism plays in two or more games at the same time’, and thus some actors will be operating within multiple identity frames. Since logics provide identities for actors, the intrusion of a new logic can have a disruptive effect (Sanders and McClellan 2014). This is evident from longitudinal empirical studies, such as the restructuring of the Alberta health care system (Reay and Hinings 2009). Here, the provincial government’s attempt to introduce a new logic of business-like health care to replace the previously dominant logic of medical professionalism was obstructed by clinicians because it reframed their identity from autonomous professional to managerial agent. The outcome was that medical professionals adapted to a hybrid identity such that they were able to deploy an appearance of complying with the managerial logic while retaining their clinical autonomy.

This example also highlights intermediating factors whose roles need to be theorized and empirically examined. These include the labour process, standards of quality or professional performance, and the regulation of particular occupational or sectoral skills (Noordegraaf 2007; Thompson and Smith 2009). These structures offer a countervailing force to the imposition of new normative frames, and deserve much greater attention in the analysis of hybrids.

## DEVELOPING THE RESEARCH AGENDA

Our analysis shows that hybridity is more than just an amalgam of sectoral characteristics. It challenges this conventional view by demonstrating that a hybrid organization arises from the existence of plural normative frames (logics) and their associated multiple actor identities. It recognizes that many public and nonprofit organizations do play in more than one game (Kraatz and Block 2008) and that as a result there is latent and overt contestation where plural institutional logics interact with actor identity mediated through professional and other structures. We hypothesize that different combinations of logics are generative of different forms of hybridity. Thus, discussions of hybridity within the public administration and nonprofit literature are liberated from the constraints of having to think of the world in terms of the state–market–community triptych. Instead, a robust theoretical platform can be introduced from which it is possible to develop, test, and analyse different models of hybridity.

Our claim is that the study of hybridity from this different ontological perspective adds significantly to scholars’ capacity to understand and explain changing forms of governance, organization, and behaviour, and also increases the possibilities for generating policy and managerial advice. ILA offers a firm theoretical base for explaining hybridity, and also brings the actor dimension into the analysis. Contests between logics are played out at an organizational level through the politics of form and structure, and at an individual level in the politics of identity. It is these properties that make the study of hybrids so interesting from a scholarly and practical point of view. It introduces questions about the process by which plural institutional logics are constructed, contested, and negotiated, the ways in which settlements are reached between them, the factors that disrupt such negotiated orders, and the consequences for the work of the organization and its relationship with members, users, and stakeholders.

So far, our discussion has concentrated on explaining *how* different elements of the model interact. The final step is to illustrate the types of variables and propositions that can be derived in order empirically to test this theory. The first variable is *normative strength*, some measure of the intensity of the prescriptions within the institutional logics applying to a particular hybrid or sector. This is of central importance because of the way hybrid organizations are located in a field of conflicting institutional logics which by definition normatively frame actors and activities, and whose symbolic content is associated with particular material resources, for example financial allocations and regulations that can authoritatively affect organizational conditions of existence. We hypothesize that the subjective appreciation of the normative strength of plural institutional logics in a particular context is an important determinant of an organization’s response. The stronger the normative environment, the more likely we are to observe segregated rather than segmented compartmentalization. This is because, as we argue above, an organization needs to be able to separate its activities in order that they clearly align with each logic (Minkoff 2002; Pache and Santos 2013).

A second variable is *actor identity*. This is a central feature of the institutional logics approach, since the normative frames supply and give meaning to actors’ roles and behaviours. Thus we may expect that as differences between the actor identities supplied by the prevailing logics increase, so the propensity for a blocked hybrid will also increase due to the difficulties individuals have in accommodating such variation (Pratt and Foreman 2000). However, this likelihood of a blocked hybrid may be moderated by our third variable – the *value commitment* of organizational members, for example in a faith organization or specialist local service provider (Smith and Sosin 2001). We hypothesize that assimilation will occur as actors seek to sustain their core practices and identity by trading off surface compliance with intruding logics for legitimacy with key stakeholders (Zilber 2002).

Finally, we hypothesize that blended hybrids are more likely to be associated with our fourth variable, *environmental turbulence*, since turbulent environments offer space for creative and innovative responses by organizations, drawing on different aspects of plural institutional logics (Binder 2007). We also hypothesize that environmental turbulence may offer social entrepreneurs a vision of entirely new organizations using a blended model as a market niche opportunity. A blended model can thus be an adaptive response to environmental turbulence of an existing organization or a new organizational model.

Developing and testing the ILA theory of hybridity has direct benefits for those working in or advising public and nonprofit organizations. It will enable them to understand more about the ways in which organizations manage plural institutional opportunities and constraints, and the strategies through which organizational sustainability can be enhanced. These management implications are particularly important where environmental turbulence places a premium on innovation and creativity in organizational design. Recent major governance innovations – public–private partnerships or charities with for-profit subsidiaries – all demonstrate that skilful management of the tensions between the different institutional logics, labour processes, and actor identities is necessary if the benefits are to be realized (Smith 2010; Saz-Carranza and Longo 2012). Further, our research should offer insights on the relationship between effective governance, broadly defined, and the successful adaptive response to environmental turbulence. So while this article has focused on theory, our ambition is that the ideas we present should ultimately be of value to those governing, working in, or engaging with public and nonprofit organizations.
